# Cryptococcus gattii: A Poseur to Behold!

**DOI:** 10.7759/cureus.28344

**Published:** 2022-08-24

**Authors:** Sabha Ahmed, Jitender Saini, M Netravathi, Poonkodi Manohar, Nagarathna Chandrashekar

**Affiliations:** 1 Neuroimaging and Interventional Radiology, National Institute of Mental Health and Neurosciences, Bangalore, IND; 2 Neurology, National Institute of Mental Health and Neurosciences, Bangalore, IND; 3 Neuropathology, National Institute of Mental Health and Neurosciences, Bangalore, IND; 4 Neuromicrobiology, National Institute of Mental Health and Neurosciences, Bangalore, IND

**Keywords:** neuroinfection, atypical presentation of sarcoidosis, immunocompetent patients, leptomeningeal dissemination, disseminated cryptococcus

## Abstract

Cryptococcosis is an invasive systemic mycosis caused by *Cryptococcus*, a genus of yeast. Causative organisms for human cryptococcosis include *Cryptococcus neoformans* and *Cryptococcus gattii*. Disease due to *C.neoformans* is conventionally seen in patients with underlying immunosuppression, whereas *C.gattii*-related infection is usually seen in immunocompetent people. The fact that the infection can occur among otherwise healthy individuals underscores the importance of having a necessary understanding of the pathophysiology and clinical and radiological presentations of the disease. We report a case of disseminated pulmonary and central nervous system (CNS) cryptococcosis in an apparently immunocompetent individual with unusual radiological findings necessitating probing for alternative diagnoses. We have attempted to supplement and revise the existing data on the radiological manifestations of *C.gattii*.

## Introduction

Cryptococcosis is an invasive systemic mycosis most commonly encountered in humans afflicted by intrinsically pathogenic encapsulated yeasts of the genus *Cryptococcus* [1]. The commonly encountered species of this genus, *Cryptococcus neoformans,* has been deemed since its discovery in 1894 and subsequent surge in the 1970s as an opportunistic pathogen inciting debilitating disseminated systemic and in particular central nervous system (CNS) mycosis in immunocompromised patients [2,3]. *Cryptococcus neoformans* is also notorious for its global domination as an opportunistic pathogen as opposed to* Cryptococcus gattii* which has made chance sporadic appearances albeit in a similar capacity in tropical nations [4]. *Cryptococcus gattii* is a newly emerging pathogen among the immunocompetent inhabitants of tropical countries with a well-documented inclination to inflict on the central nervous system/lungs or both [4].

The most commonly encountered and dreaded form of clinical presentation in infections incited by either agent is meningoencephalitis on account of the neurotropic nature of both the offending agents [4]. However, CNS dissemination of *C. gattii* is preceded by inhalation of infective spores that harbor within the lung parenchyma precipitating dormant/active lung infection [4,5].

The non-specificity of clinical presentation in immunocompetent hosts, in particular with underlying dormant/latent pulmonary disease as described in our case report, often poses a diagnostic dilemma to the index physician at presentation. Further, the atypical imaging findings as illustrated in our case are often a hurdle for a conclusive tailored set of differentials. We thus report a rare case of disseminated pulmonary and central nervous system (CNS) cryptococcosis caused by *C. gattii* with unconventional clinical and imaging phenotype in an immunocompetent adult to append the existing literature on the radiological phenotype of this disease.

## Case presentation

An apparently healthy 43-year-old male, farmer by occupation, without any comorbidities was admitted with six months history of new-onset headaches that were holocranial, throbbing in character, mild to moderate in severity, and was not associated with nausea or vomiting at the onset. The headaches worsened 15 days prior to admission with their intensity becoming severe and associated with vomiting. The patient also had a single episode of left focal seizure with impaired awareness 15 days before presentation. He was a non-smoker and occasionally consumed alcohol with no history of injectable drug abuse/high-risk sexual behavior. At presentation, he was afebrile, and his systemic examination was normal. The patient was fully conscious and oriented at index presentation. Neurological examination did not reveal any signs of meningism, cranial nerve, or other focal neurological deficits.

**Figure 1:**
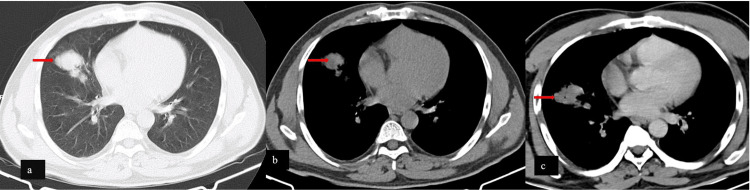
Cross-sectional imaging of the chest The lung window of high-resolution computed tomography shows: (a) a multilobulated relatively smoothly marginated lesion simulating the course of bronchioles in the right middle lobe, and (b) axial sections of soft tissue. Contrast-enhanced computed tomography reveals (c) the low-density intralesional content with non-enhancement.

On account of the ongoing COVID-19 pandemic and the frequent occurrence of CNS manifestations even in the setting of asymptomatic pulmonary infliction, a chest radiograph was obtained. A well-defined opacity was discernible in the right lower zone which prompted cross-sectional evaluation. Low dose high resolution computed tomography (HRCT) and a contrast-enhanced computed tomography of the chest revealed a well-defined smoothly marginated multilobulated opacity in the periphery of the right middle lobe with low-density contents which didn’t show any significant contrast enhancement (Figure 1 b and c). A peculiar orientation simulating the track of secondary bronchioles was identified (Figure 1 a). No significant lymphadenopathy or bronchus cut-off was seen.

**Figure 2:**
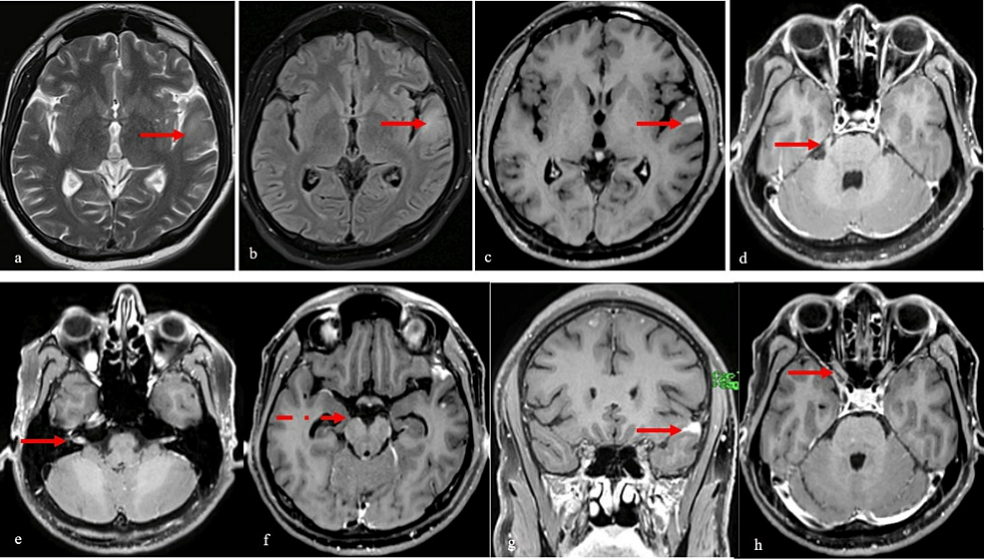
MRI of the brain Axial T2WI (a) demonstrates a discrete focus of hyperintensity with the corresponding hyperintensity on axial FLAIR (b) and non-enhancement of the intraparenchymal focus with vivid nodular gyriform enhancement of the overlying gyri of axial post-contrast T1WI (c). Nodular perineural enhancement of the cisternal segments of bilateral trigeminal (arrow in d), meatal segments of the bilateral facial (arrow in e), the pial surface of the right oculomotor nerve (dashed arrow in f), and prechiasmatic and intracanalicular segments of bilateral optic nerves (arrow in h) are identified on multi-level axial sections of post-contrast T1WI (d,e,f, and h, respectively). Multiple foci of punctate and gyriform nodular enhancement are identified in bilateral cerebral hemispheres on coronal post-contrast T1WI (g). T1WI: T1 weighted image, T2WI: T2 weighted image, FLAIR: Fluid-attenuated inversion recovery

Concurrently, a contrast-enhanced MRI of the brain was performed due to the presence of headache and one episode of seizure. A non-enhancing focus of T2-weighted-fluid-attenuated inversion recovery (T2/FLAIR) hyperintensity (Figure 2 a, b, and c) was identified in the left superior temporal gyrus. Multifocal areas of vivid nodular gyral enhancement (Figure 2 g) were seen in bilateral cerebral hemispheres. In addition, pial-based enhancement of the right oculomotor nerve (Figure 2 f) and nodular perineural enhancement of the meatal segments of bilateral facial (Figure 2 e), transitional segments of bilateral trigeminal (Figure 2 d), prechiasmatic, and intracanalicular segments of bilateral optic nerves (Figure 2 h) were also observed.

The amalgamation of pulmonary and CNS imaging findings prompted a differential of primary lung mass with leptomeningeal carcinomatosis. However, considering the atypical appearance of the lung mass, the second possibility of sarcoidosis was also kept. The diagnostic dilemma prompted a histopathologic and laboratory correlation.

Preliminary investigations revealed normal kidney and liver function tests, serum electrolytes, and a negative antinuclear antigen (ANA) profile. A mildly elevated neutrophil count of 77.4% with normal levels of the remaining complete blood count (CBC) parameters was found. Serology comprising of Hepatitis B, C, and human immunodeficiency virus (HIV) was negative. Cerebrospinal fluid (CSF) revealed an opening pressure of 140 mm Hg, with a cell count of 136 cells/cumm with 75% lymphocytes, elevated protein levels (121.4 mg/dl), and mildly low sugars (CSF sugar 49 mg/dl with corresponding blood sugars of 96 mg/dl). The CSF cytospin yielded moderately cellular (mature lymphocytes>neutrophils) cytomorphology, and there were no atypical cells. The CSF was found to have India ink positivity and rapid cryptococcus species capsular antigen was positive by the lateral flow method. Gram staining of CSF as well as the CSF venereal disease research laboratory (VDRL) test, cartridge-based nucleic acid amplification test (CBNAAT), and *Brucella*-polymerase chain reaction (PCR) were negative. 

The CSF sample sent to the neuromicrobiology laboratory was inoculated on sabouraud dextrose agar (SDA) and incubated aerobically at 25° C to 30° C. The growth on SDA showed cream-colored smooth, mucoid, yeast-like colonies. Microscopic examination of the colony revealed spherical budding yeast cells measuring 4 µm to 6 µm in diameter, surrounded by a capsule morphologically resembling the *Cryptococcus *species complex. The yeast was identified as *C. gattii *by an automated mass spectrometry microbial identification system that uses matrix-assisted laser desorption ionization time-of-flight (MALDI-TOF) technology which works based on a characteristic protein spectra extracted from whole cells where the series of peaks detected are sorted by mass and intensity and thereby produces a highly discriminatory identification of a pure culture in five to 20 minutes at minimal cost for optimal patient care decisions.

**Figure 3:**
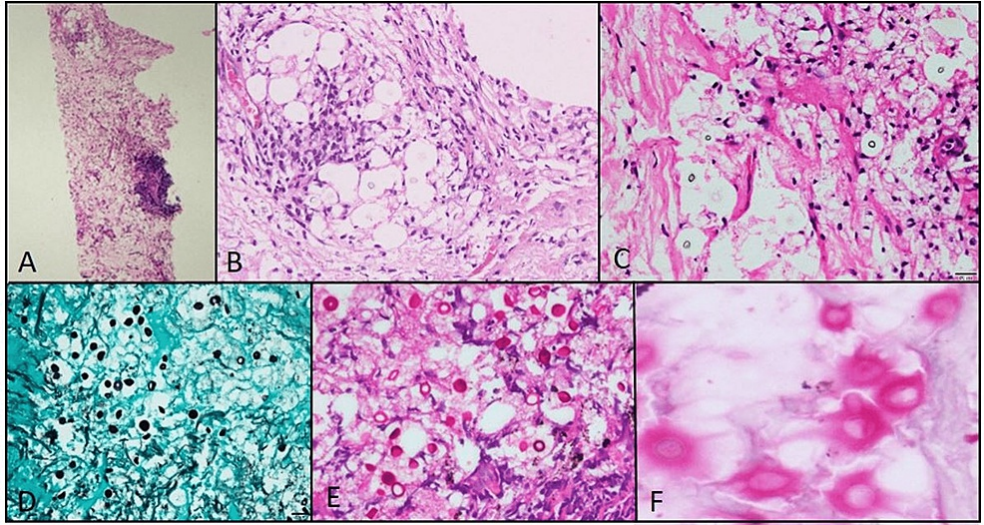
Histopathological images of biopsied lung lesion A: Core of lung biopsy shows focal dense lymphohistiocytic inflammation, B: Higher magnification shows lymphohistiocytic inflammation with an ill-formed granuloma, C: Refractile round to oval cryptococcus yeasts with thin cell wall surrounded by a pale non-staining halo, D: The organism’s cell wall is highlighted in Gomori’s methenamine silver stain, E: The organism’s cell wall highlighted in periodic acid-Schiff stain, F: Thick mucopolysaccharide capsule is stained in the mucicarmine stain Original  magnification: (A)x40; (B)x100; (C), (D) and (E)x200; (F)x400

The lung mass was also taken up for percutaneous biopsy. Histopathology revealed scattered round to oval encapsulated yeasts resembling cryptococcus among bits of lung parenchyma. The stroma showed lymphohistiocytic inflammation, focal lymphoid aggregates, an occasional poorly formed granuloma, and focal necrosis. There were no giant cells. Periodic acid-Schiff (PAS) and Gomori methenamine silver (GMS) staining highlighted the cell walls of the yeast forms. No atypical cells were detected (Figure 3).

A conclusive diagnosis of pulmonary and CNS cryptococcosis by *C. gattii* was made. An effort was made to evaluate other causes of cell-mediated immunodeficiency owing to their known association with this infection. The patient had a cluster of differentiation 4 (CD4) count of 448 cells/mm^3^ with marginally elevated immunoglobulin E (IgE) levels. Hence, no evidence of an underlying immunosuppressed state was seen in our patient.

The preliminary induction therapy consisted of an intravenous injection of amphotericin B and flucytosine. However, on account of unresponsiveness to this combination and inability to run the drug sensitivity panel, fluconazole and voriconazole were initiated. A follow-up CSF analysis two weeks later tested negative for India ink and cryptococcal antigen implying a good response to therapy. Repeat HRCT chest did not reveal any significant difference in the size and density of the lung mass. The patient was discharged asymptomatic on consolidation therapy with flucytosine, fluconazole, and voriconazole.

## Discussion

Disseminated cryptococcosis due to *C. gattii *has emerged as an important cause of systemic mycosis among both immunocompetent as well as immunocompromised hosts in the Indian subcontinent. Although a few cases of concurrent pulmonary and CNS cryptococcosis have been described in Asia, the occurrence of the same in India is rather sporadic and is yet to find a hold in warranting the attention of clinicians [6]. The atypical radiological findings described in our case merit a re-assessment of the pre-existing literature on the imaging phenotypes of this entity addressing the diagnostic dilemma such an imaging presentation could entail.

Although serological evidence of cryptococcal infection is common in immunocompetent individuals, cryptococcal disease is relatively rare in the absence of an immunocompromised state. *Cryptococcus neoformans* is well known for disease causation in patients with underlying immunosuppression such as advanced HIV disease, solid organ transplant recipients, patients with hematological malignancies, or those on chronic glucocorticoid use [2,3]. However, despite sharing a common species complex with *C. neoformans, C. gattii* exhibits different ecological niches and molecular karyotyping and is well described for its affinity for disease causation in immunocompetent hosts [5]. Among the four molecular subtypes of *C. gattii*, VG1 is the predominant variant described in Asia with a known chronology of pulmonary affliction followed by CNS dissemination in immunocompetent hosts [7]. Our patient did not have any underlying disease that could potentially have been causative of his disease manifestation. However, being a farmer by occupation he was possibly exposed to inhaling these fungal spores given the natural reservoir of this pathogen in plant debris and soil in addition to being associated with numerous tree species, in particular, the *Eucalyptus* [8]. The tropical climatic conditions coupled with the prevalence of eucalyptus trees in South India possibly aid the survival of *C. gattii *in this part of the Indian subcontinent [8]. Given that the cryptococcal infection is acquired through inhalation of infectious aerosols, the pulmonary defense mechanisms are believed to be highly effective in containing the fungus. The prolonged harboring of the fungus inside the respiratory system has been postulated to result in the formation of granulomas. While extrapulmonary dissemination is an expected event in immunocompromised patients, what causes such a phenomena in immunocompetent subjects is still not well understood. Studies have shown that defective neutrophil recruitment and lymphopenia can initiate such a disease cascade in immunocompetent individuals also [9,10].

The pulmonary cryptococcoma in our patient was asymptomatic. Such presentation of a solitary pulmonary cryptococcoma as in our case is one of the rare manifestations of this disease entity. The smooth borders, low attenuation, and predominant non-enhancement of the mass are well-documented features of pulmonary cryptococcomas [11]. However, the size, absence of ancillary infective parenchymal sine que non, and solitary nature of the mass did pose an initial diagnostic dilemma that necessitated biopsy for histological confirmation of the mass.

Radiologically, CNS cryptococcosis in an immunocompetent host appears in the form of meningitis with or without vasculitis, clustered cysts, or fine nodules in a background of meningoencephalitis, choroid plexitis, and solitary large arachnoid cysts [12,13]. The solitary CNS cryptococcomas with the suggestion of minimal inflammatory insult detected in our study could be postulated as a consequence of parenchymal invasion from the accumulation of copious amounts of fungal organisms in the cortical-subcortical white matter via dissemination through the parenchymal microvasculature/perivascular connection to subarachnoid spaces [14].

While cryptococcomas are commonplace in the radiological atlas of *C. gattii*, the distinct nodular enhancement of the cranial nerves as illustrated in our case prompted a differential of neurosarcoidosis and leptomeningeal metastases assuming a primary lung neoplasm, a rather common distraction in the few case reports of concurrent lung and CNS *C. gattii *infection that has been described in the literature [15,16]. The pathophysiology of cranial nerve enhancement rests on a gamut of insults ranging from inflammation, neoplasm, autoimmune, radiation, and ischemia, among others as instigating factors culminating in the disruption of the blood-brain barrier and concomitant abnormal enhancement [17]. Saremi et al. also described a case of perineural enhancement of unilateral optic nerve in non-HIV hosts [17]. However, to the best of our knowledge, the florid cranial nerve enhancement as illustrated in our case is yet to be reported and explored.

Few radiological pointers that could have prompted a differential of cryptococcus in our case are the distinct non- enhancing T2/FLAIR hyperintensity in the left superior temporal gyrus, an imaging correlate of cerebral cryptococcoma, and the gyriform nodular leptomeningeal deposits. These findings are touted as a commonality in cryptococcosis among immunocompetent subjects as described by Chen et al. [12].

The preliminary reports on *C. gattii* infection in immunocompetent hosts came from Australia and New Guinea [14]. The Australian case series elaborated on the common clinical abnormalities encountered, namely multiple cranial nerve deficits, seizures, cerebellar signs, hearing loss, and abnormal mental status. Impressive data on the prevalence of raised intracranial pressure (ICP) in CNS *C. gattii* insult is also existent in literature [9,18,19]. Our case is peculiar in not having a clinical correlate of cranial nerve involvement and meningism despite the remarkable radiological manifestations of the same.

While much has been published regarding the availability of rapid tests for early initiation of medical therapy for this disease entity, our case illustrates the need to revise the radiological presentations of disseminated *C. gattii* infection. Furthermore, the good treatment response in our case, as opposed to the poor prognosis deemed in cases that have positive blood culture and concurrent pulmonary and lung changes, reiterates the need for early diagnosis and timely initiation of therapy in such patients.

## Conclusions

In conclusion, we reiterate the peculiar radiological manifestation of vivid cranial nerve enhancement in a histopathologically proven case of *C. gattii *thereby emphasizing the need to reconsider our understanding and differentials of disseminated cranial nerve enhancement. The remarkable treatment response to medical therapy as in our case further establishes the ground for a high index of clinical as well as radiological suspicion in patients with concurrent pulmonary and CNS features. The need to expand the turf of *C. gattii* imaging correlates is thereby recommended.
